# Indents

**Published:** 1910-07-15

**Authors:** 


### INDENTS.

**☞ Brief Articles of Technical or Scientific Value will receive notice and comment. Contributions invited.**

*On the Treatment of Aphthae with Local Applications of Formol.* Dr. Dessirier of *Lyon*, preconises Formol in the treatment of aphthae. The advantage of this treatment is to rapidly heal and to bring upon prompt cicatrization of aphthae.

This treatment consists of a local application with a peldget of cotton saturated with a 40 per cent solution of formol, taking care to previously dry the ulceration before applying the formol solution.

The first application will cause a sharp pain which will soon pass away and will not return. This anesthetic action of formol is quite remarkable, the movements of the lips, speech, and mastication will be performed with much more ease after the first treatment. Very few applications will effect a cure. In order to cover the unpleasant taste of formol, a few drops of spirits of menthol may be added to the solution.—*American Journal.*

*Insulating Cement.* Two parts of Grecian pitch and one part of well-calcined gypsum are melted together. As long as the cement is warm it can be kneaded and worked plastically; when cold it can be turned in the lathe and polished.—*Bur.*

*Inlay Casting, A Flux for.* When the gold is fused and almost to the boiling point, a little dust of borax will clean it and make it boil quickly, and a good sharp casting will be the result. Without the borax I may force my heat much longer, and think that the gold is properly fluid, but a large percentage of cases will fail to give a good sharp casting. Do not use the borax until the gold has fused and is covering the sprue opening, for there is a possibility of the borax closing the opening and preventing the gold reaching the mold. I use borax and boracic acid, equal parts, as a flux.—S. E. Johnson, D.D.S., *Western Dental Journal*, February, 1910.

*Pain of Teeth Extraction Fatal.* Never rallying from the shock caused by the extraction of two teeth, John Nequist, 55 years old, of Whitehall, died in Muskegon, February 8.

Nequist became delirious in the dentist's office after having his teeth pulled recently and went from delirium into a paralytic state. Physicians say he had a weak heart and the shock attendant upon the pain of having the teeth removed caused paralysis.—*Daily Press.*

*Sterilizer, Inexpensive for Dental Instruments.* A large mouth, ground stoppered glass jar such as confectioners use for displaying candies, makes an efficient formalin sterilizer. Lay the jar on its side and fit to its lower side a pad of blotting paper upon which to lay instruments. In the hollow of the stopper fit another pad to be well saturated with formalin. Lay the instruments in the jar, put the stopper in place, and the sterilizer is in operation. It will be found very convenient for sterilizing instruments and appliances liable to be injured by boiling.—S. C. Herrick, D.D.S., Russell, Kansas.

